# Experiences of bullying and harassment, including sexual harassment, amongst ENT trainees in the UK: survey findings

**DOI:** 10.1017/S0022215125103587

**Published:** 2026-01

**Authors:** Vanessa Baxter, Tharsika Myuran, Winifred Eboh, Reza Majdzadeh, Frederick Green

**Affiliations:** 1 School of Health & Social Care, University of Essex, Essex, UK; 2 Association of Otolaryngologists in Training, UK; 3 Institute for Health and Care Improvement, York St John University, York, UK

**Keywords:** otolaryngologist, traineeship, bullying, harassment, sexual harassment

## Abstract

**Objectives:**

The Association of Otolaryngologists in Training wanted to assess the experiences of bullying, harassment and raising concerns in their otolaryngology posts.

**Methods:**

An online survey of otolaryngology trainees, with 190 responses out of 350 targeted, included questions on bullying and harassment.

**Results:**

Many respondents had experienced or witnessed a range of bullying, harassment and sexual harassment behaviours, including: unrealistic expectations about workload, responsibilities or level of competence; inadequate or absent supervision; and undervaluing someone’s contribution (in their presence or otherwise). However, very few (5 per cent or less) had reported them. Twenty-one per cent would not feel confident in reporting bullying/harassment or sexual harassment problems, and 40 per cent do not feel safe raising concerns. Just 10 per cent said the existing reporting mechanisms are sufficient.

**Conclusion:**

A number of initiatives have been introduced recently in the UK to address bullying and harassment within the medical workplace, but there is still potential for further development.

## Introduction

The Association of Otolaryngologists in Training (AOT) represents all ENT and Head and Neck Surgery trainees in the UK. It is independent and is run by trainees, for trainees. There are approximately two thousand ENT resident doctors of all grades in the UK, including registrars in training to become consultants, of whom some 350 are members of the AOT.

The AOT undertook a survey of ENT trainees in 2022 to gather information on their experiences of bullying and harassment, raising concerns and trainee wellbeing. They commissioned the University of Essex to run the survey, analyse responses and report on its findings.

It is estimated that bullying and other abuse cost the National Health Service (NHS) in England at least £ 2.28 bn annually through sickness absence, employee turnover and lost productivity.^1^ Bullying, harassment and sexual harassment have a negative effect on the wellbeing and productivity of trainee doctors and surgeons.

The bullying and harassment of doctors within healthcare settings appears to be prevalent across multiple countries. One systematic review^2^ found that 63 per cent of 29 980 surgical residents from 25 studies had experienced bullying, 29 per cent had experienced harassment, and 27 per cent had experienced sexual harassment. Residents who were female in gender reported experiencing all of these behaviours more often. The most common perpetrators were attending surgeons, followed by senior co-residents. Another systematic review^3^ of studies conducted in medical settings where targets were consultants or trainees, identified men as the most common perpetrators (67 per cent of 4722 respondents in 5 studies), while women were the most common targets (56 per cent of 15 246 respondents in 27 studies). Consultants were reported as the most common perpetrators (54 per cent of 15 868 respondents in 31 studies).

Both of these reviews found that only a minority of targets (under a third) reported the behaviour – with just over half stating that this was due to the fear of reprisals.^2^ Over half of those who reported their experiences had had a negative experience of doing so.^3^ The facilitators of bullying included not enforcing institutional policies (reported in 13 studies), the normalisation of bullying (10 studies) and hierarchical power structures (7 studies).^3^

Experiences of bullying and harassment decrease with age, with those under 30 being more likely to experience these issues.^4^^,^^5^ Ethnicity – white *vs* non-white ethnicities – and nationality –doctors from Europe compared to non-European doctors – are other factors that have been found to be associated with workplace bullying.^5^

There has been little research into the true scale of the sexual harassment problem in medicine, but one systematic review^6^ concluded that nearly 60 per cent of medical students and trainees of all grades experience harassment or discrimination of some kind during their training, with females being targeted more than males. Consultants were the most common perpetrators, and sexual harassment was the most frequent form of abuse.^6^ The majority of sexual harassment incidents appear to go unreported, mainly through fear of the consequences on women’s careers of reporting harassment or whistle-blowing.^7^ One qualitative study found that women expect their experiences of abuse to be disbelieved or dismissed as exaggeration, or blamed on their own appearance and behaviour, or they are told it is an ‘understood condition’ within their specialty. The effects on women, both personally and professionally, may be severe and enduring.^8^

## Aim of the study

The AOT wanted to assess the experiences of bullying, harassment and raising concerns in their ENT posts.

## Materials and methods

### Methodology

The AOT drafted an online survey that the University then reviewed and suggested additions to/minor amendments. This included questions on bullying and harassment, sexual harassment and raising concerns. Demographics, including UK region, age, level of training, gender, sexual orientation, ethnicity and religion were also recorded.

### Recruitment

Participation was voluntary, and an online survey link was circulated to AOT members via email. At the end of the survey, links to sources of support and information were provided, including advice on whistle-blowing with the NHS, and both the British Medical Association (BMA) and NHS Employers Guidance on Harassment and Bullying.

### Participants

The survey was live between October and December 2022, and 190 responses were received, a 54.3 per cent response rate (out of an estimated AOT trainee population of 350). This is on a par with the average response rate of 53.3 per cent ± 24.5 per cent (mean ± SD) of 1746 online surveys of health care professionals.^9^

Of the 190 respondents, 81 per cent were registrars, 44 per cent were male (*n* = 88), 41 per cent were female (*n* = 82), and the remainder were non-binary or preferred not to say. Eighty-six per cent (*n* = 153) were straight/heterosexual, 3 per cent (*n* = 6) were gay or lesbian, 3 per cent (*n* = 5) were bisexual, and the remainder preferred not to say. Seventy-nine per cent of respondents (*n* = 147) were aged 30–39.

Respondents were asked whether they work less than full-time, with 67 per cent saying that they do not. Seventeen per cent of females said ‘Yes, under category 1 (disability/ill health/caring responsibilities)’ (3 per cent of males), and 30 per cent of females said ‘No – but would like to’ (15 per cent of males).

### Data analysis

Survey responses were analysed within Excel (Microsoft, Redmond, Washington, USA) and SPSS (IBM, Armonk, New York, USA).

The statistical analysis method includes both descriptive and analytical methods. The descriptive one utilises categories of bullying and harassment to demonstrate the respondents’ situation, including non-respondents. Numbers and percentages are reported for each category to emphasise the importance of exposure to harassment.

Descriptive statistics were used to analyse categorical variables, which utilises scores from the questionnaire responses and reports the mean, standard deviation and standard error. Since the responses were categories and had not normally distributed, means between demographic groups were compared using the non-parametric Kruskal–Wallis for multiple group comparisons and Mann–Whitney for two groups, with significance levels considered at (*p* = 0.05). Due to the number of statistical tests for pairwise comparisons, significance values were adjusted using the Bonferroni method.

To examine the difference in bullying and harassment experiences between women and men, the analysis utilised the percentage of each group to describe and calculate odds ratios for the effect size. The 95 per cent confidence interval was estimated for the odds ratio, and the gender differences were statistically evaluated using chi-square.

### Limitations

An online survey has several limitations. Firstly, the response rate cannot be exactly determined, since it is not possible to know how many trainees viewed the email invitation. A lower response rate reduces the generalisability of the findings. Although the total ENT trainee population is unknown, 190 responses out of an AOT membership of 350 gives an estimated response rate of 54.3 per cent.

Secondly, there may be a sampling bias amongst responders, since they are self-selecting, because participation was voluntary. This may further affect generalisability. This is an interesting point since many healthcare surveys of professionals (e.g. General Medical Council (GMC), Intercollegiate Surgical Curriculum Programme (ISCP)) are mandatory.

## Results and analysis

### Bullying and harassment

For 77 per cent of respondents (*n* = 109), their workplace makes it clear that unsupportive language and behaviour are not acceptable (e.g. condescending or intimidating language, ridicule, overly familiar behaviour, jokes/banter that stereotype people or focus on their appearance or characteristics). However, 13 per cent (*n* = 20) disagreed, and 9 per cent (*n* = 13) disagreed strongly.

When questioned on specific bullying or harassment behaviours within the previous six months, 33 per cent of respondents (*n* = 51) reported that they had experienced unrealistic expectations about workload, responsibilities or level of competence, and 22 per cent (*n* = 33) had witnessed this. Twenty-five per cent (*n* = 39) had experienced inadequate or absent supervision, while 16 per cent (*n* = 24) had witnessed this. Twenty-five per cent (*n* = 38) had experienced undervaluing someone’s contribution (in their presence or otherwise), while 18 per cent (*n* = 28) had witnessed this. Very few respondents (under 5 per cent for bullying and harassment and 1 per cent or less for sexual harassment) had reported any of these behaviours (Table 1).

**Table 1.** Experiences of bullying and harassment by all respondents

	I have experienced this Frequency (*n* = 153)	I have witnessed this Frequency (*n* = 153)
Undermining someone’s role, e.g. criticism in front of patients or other staff	29 (19%)	34 (22%)
Persistent or excessive negative feedback; unsubstantiated allegations	26 (17%)	24 (16%)
Asking trainees to perform tasks they have not been trained to do	18 (12%)	14 (9%)
Asking trainees to work unpaid shifts	23 (15%)	10 (7%)
Undervaluing someone’s contribution (in their presence or otherwise)	38 (25%)	28 (18%)
Unrealistic expectations about workload, responsibilities or level of competence	51 (33%)	33 (22%)
A member of staff shouting or swearing at someone	26 (17%)	23 (15%)
Excluding, devaluing or ignoring an individual on purpose	25 (16%)	19 (12%)
Inadequate or absent supervision	39 (25%)	24 (16%)
Belittling or marginalisation of trainees by senior staff from other professional groups	26 (17%)	22 (14%)
Bullying of trainees by other staff pursuing targets	28 (18%)	25 (16%)
Abusing position of seniority to make demands	34 (22%)	24 (16%)
Abusing position of seniority in job selection/loss of job opportunity	20 (13%)	19 (12%)

Analysis of respondents’ experiences of bullying and harassment by gender shows two statistically significant differences between female (*n* = 82) and male respondents (*n* = 88). Females were 2.6 times more likely than males (*p* = .0102) to say that they had experienced unrealistic expectations about workload, and 2.8 times more likely (*p* = .0146) to say they had experienced inadequate or absent supervision (Table 2).

**Table 2.** Experiences of bullying and harassment by gender

I have experienced this	Total responses*	Females	Males	*p* value
Undermining someone’s role, e.g. criticism in front of patients or other staff	129	16 (26%)	10 (15%)	0.2295
Persistent or excessive negative feedback; unsubstantiated allegations	126	13 (21%)	11 (17%)	0.5307
Asking trainees to perform tasks they have not been trained to do	126	6 (10%)	8 (12%)	0.6591
Asking trainees to work unpaid shifts	125	11 (18%)	10 (15%)	0.6595
Undervaluing someone’s contribution (in their presence or otherwise)	127	20 (33%)	15 (23%)	0.2049
Unrealistic expectations about workload, responsibilities or level of competence	125	29 (48%)	17 (26%)	0.0102
A member of staff shouting or swearing at someone	123	11 (19%)	11 (17%)	0.8332
Excluding, devaluing or ignoring an individual on purpose	126	15 (25%)	8 (12%)	0.0745
Inadequate or absent supervision	126	22 (36%)	11 (17%)	0.0146
Belittling or marginalisation of trainees by senior staff from other professional groups	126	13 (21%)	10 (15%)	0.3894
Bullying of trainees by other staff pursuing targets	125	16 (26%)	11 (17%)	0.2195
Abusing position of seniority to make demands	125	16 (26%)	14 (22%)	0.5688
Abusing position of seniority in job selection/loss of job opportunity	126	6 (10%)	8 (12%)	0.6591

* (Where gender was reported by the respondent)

### Sexual harassment

Eighteen per cent of respondents (*n* = 29) had experienced or witnessed sexual harassment behaviours at work in the last six months in the form of comments on physical appearance. Sixteen per cent (*n* = 26) had experienced or witnessed intrusive comments about personal life, and 13 per cent (*n* = 22) had experienced or witnessed lewd comments. Just 1 per cent had reported any of the sexual harassment behaviours (Table 3).

**Table 3.** Experiences of sexual harassment by all respondents

	I have experienced this Frequency (*n* = 163)	I have witnessed this Frequency (*n* = 163)
Ogling/staring	8 (5%)	3 (2%)
Lewd comments	10 (6%)	12 (7%)
Intrusive comments about personal life	15 (9%)	11 (7%)
Comments on physical appearance	17 (10%)	12 (7%)
Unsolicited texts, emails, pictures, social media posts	4 (2%)	4 (2%)
Violating personal space: patting, grabbing, caressing, hugs, kisses	3 (2%)	3 (2%)
Physical assault	1 (1%)	1 (1%)
Sexual assault	1 (1%)	2 (1%)
Rape	1 (1%)	0 (0%)

Female respondents (*n* = 82) were significantly more likely than males (*n* = 88) to say they had experienced intrusive comments about their personal life (*p* = .0005) or comments on their physical appearance (*p* = .0423). All of the other behaviours (apart from unsolicited texts/emails/pictures/social media posts) were only experienced by female respondents, not by male respondents (Table 4).

**Table 4.** Experiences of sexual harassment by gender

I have experienced this	Total responses*	Females	Males	*p* value
Ogling/staring	124	6 (10%)	0 (0%)	0.0107
Lewd comments	124	5 (8%)	3 (5%)	0.4364
Intrusive comments about personal life	125	13 (21%)	1 (2%)	0.0005
Comments on physical appearance	123	11 (18%)	4 (6%)	0.0423
Unsolicited texts, emails, pictures, social media posts	124	3 (5%)	1 (2%)	0.2940
Violating personal space: patting, grabbing, caressing, hugs, kisses	123	3 (5%)	0 (0%)	0.0724
Physical assault	124	1 (2%)	0 (0%)	0.3075
Sexual assault	124	2 (3%)	0 (0%)	0.1474
Rape	124	1 (2%)	0 (0%)	0.3075

* (Where gender was reported by the respondent)

When asked to whom respondents would feel confident reporting bullying or sexual harassment behaviours, the main replies were educational or clinical supervisors (ES or CS) or training programme directors (TPD). However, 22 per cent (*n* = 31) would not feel confident in reporting bullying problems, and 18 per cent (*n* = 24) would not feel confident in reporting incidents of sexual harassment. The main reasons why respondents would not feel confident about reporting these problems were that it is unlikely that any action will be taken or no action has been taken in the past, and due to the fear of repercussions or being seen as a difficult trainee, including doubts about the confidentiality of the process.

*‘Consultants/supervisors know about it, witness it, trainees report it, and nothing is done about it.’ (Female respondent)*

*‘I discussed the most serious incident of bullying with the TPD, who although sympathetic, did not give a confidence-inspiring response, suggesting I apologise that the perpetrator had felt compelled to swear and shout at me and offer to move on professionally for the sake of patients, citing the likelihood that a formal complaint would more likely impact future employers’ perception of me than result in any action against the consultant responsible for the bullying.’ (Male respondent)*

*‘No secure way to report bullying and having witnessed a trainee be collectively attacked after reported (valid) bullying and undermining behaviour, I know consultants and managers will always stick together.’ (Female respondent)*

*‘My specialty is small. Remaining anonymous is near impossible.’ (Female respondent)*

### Reporting concerns

Forty-one per cent of respondents were aware of mechanisms for reporting concerns about bullying, harassment and sexual harassment, but 35 per cent were not, and 24 per cent were unsure. While 52 per cent of respondents (*n* = 71) reported that they feel safe about raising concerns about bullying or harassment, 38 per cent (*n* = 53) do not feel safe. For the 53 respondents who did not feel safe raising concerns, the biggest barriers to reporting any inappropriate behaviour that respondents have witnessed or experienced are not wanting to potentially harm their career (94 per cent, *n* = 50), feeling that nothing will change (77 per cent, *n* = 41) and not wanting to be seen as a whistle-blower (64 per cent, *n* = 34). A quarter (25 per cent, *n* = 13) reported that they have been warned against doing so.

*‘There is a culture of not escalating or investigating bullying and harassment concerns, even when they are reported. I have been told that formally reporting these concerns would adversely affect my career.’ (Respondent who did not specify gender)*

*‘There is a misogynistic culture that is rampant in the NHS and hidden in plain sight – the changes have to come from within, and a wider debate in society is needed to address these issues.’ (Male respondent)*

*‘NHS trusts and training bodies act to cover up sexual abuse and blame the trainee, e.g. accuse them of lying or not being able to cope with training.’ (Female respondent)*

Just 10 per cent of respondents (*n* = 15) stated that existing mechanisms of reporting are sufficient. The most popular features that respondents would like to see in a confidential reporting system were protection of the identity of those raising concerns, clustering units to preserve anonymity and the use of investigators from other specialties, followed by logging incidents to retain records for future reference.

*‘Only way is an anonymous validated reporting system. Otherwise, people start posting anon posts on websites targeting 1 Consultant. This is also wrong. The accused should also be protected and, if valid, given training and counselling.’ (Female respondent)*

*‘Reporting has to be normalised by consultants and encouraged. Consultants’ language and attitudes need to change so that this empowers juniors to bring up concerns. Consultants talking, calling out inappropriate behaviour and acting as role models. Only when trainees know it will be taken seriously will they feel confident reporting won’t impact negatively on their career. There is a lot of “in my day” we had to work in terrible conditions, therefore juniors shouldn’t complain.’ (Female respondent)*

*‘By creating a system where a person who raises concerns feels safe, anonymous, protected and absolutely certain that it cannot be traced back to the individual and affect future placements or career prospects.’ (Female respondent)*

## Discussion

In recent years, there have been various surveys and reports identifying the existence of bullying, harassment and misconduct in surgical training in the UK and other countries, with a number of specific recommendations made by different medical organisations.

Fifty-five per cent of the 58 respondents to a survey in 2023 by the Association of Surgeons in Training (ASiT)^10^ in the UK had experienced bullying, while 78 per cent had witnessed it, but 67 per cent had not reported it. ASiT subsequently identified a lack of awareness of pathways for raising concerns (apart from reporting to CS and ES if in a training post), and that the most common reasons for not speaking up were that ‘nothing will change’ or ‘I will be considered a troublemaker’.

It is known that bullying and harassment are linked to poor wellbeing and mental health, as bullying increases the risk of psychological distress and mental health issues among doctors.^11^^,^^12^ Research commissioned by the BMA in 2020^13^ identified five potential groups of risk factors for poor wellbeing, one of which was interpersonal factors derived from doctors’ relationships with their peers, including issues related to hierarchy and bullying.

The BMA report in 2020 also recommended a long-term strategy for supporting the mental health and physical health of doctors and staff, including a number of points that are pertinent to the findings from this survey. Support should be inclusive, accessible, and meet users’ needs, taking into consideration the diversity of staff and their differing experiences of mental health: this needs to include groups such as doctors with a disability and International Medical Graduates (IMGs) (who are new to medical practice in the UK) who face additional barriers to accessing support. Another recommendation is for the active encouragement of peer support and mentoring, which could include buddying up experienced and inexperienced workers and setting up Schwartz rounds or Balint groups.

Recommendations made by the Academy of Medical Royal Colleges’ Academy Trainee Doctors’ Group (ATDG), in response to the UK Parliament Health and Social Care Committee’s Inquiry into Workforce,^14^ also included one around the provision of wellbeing/support resources to tackle rising levels of burnout, with continuing work to remove incidences of bullying and harassment within training.

The findings from this study indicate that while many ENT trainees have experienced bullying and harassment, they are unlikely to report this, with comments reflecting an unwillingness to report concerns to ‘the hierarchy’ (consultants and managers). A safe reporting environment is therefore needed, underpinned by counselling and support services as required. Non-UK trained staff should be informed about such services, and all trainees should be made aware of the voluntary organisations that give support and that are independent of NHS Trusts. Any reporting process must guarantee anonymity, confidentiality and protection for targets and reporting staff, although this can be problematic as anonymity is hard to maintain when following up on a reported problem that is a criminal act. There needs to be a way that those targeted can report mistreatment and abuse, but the system also needs to provide support for the targets, and that support system should be anonymised.

The body of evidence has resulted in a number of initiatives within the UK to combat and eradicate bullying and harassment within surgical and other healthcare working environments. In 2017, the BMA launched a project on workplace bullying and harassment to improve support for individual doctors who experience it, work with NHS organisations and partners to address it, and raise awareness of bullying and harassment across the profession. An Alliance Against Bullying, Undermining and Harassment in the NHS^15^ was formed in 2019 to share ideas and enact interventions across the whole of the NHS. In 2022, the GMC set out plans to include a new duty for doctors that would require them to act, or help others to act, if they observe workplace bullying, harassment, or discrimination. They would also be asked to adopt a zero-tolerance approach to sexual harassment. These proposals have now been included within the Good Medical Practice,^16^ which sets out the professional values, knowledge and behaviours expected of doctors working in the UK.

The Royal College of Surgeons of Edinburgh (RCSEd) launched the #LetsRemoveIt campaign^17^ in 2017 to tackle bullying and undermining within the surgical workforce, and since 2024, the campaign has focused on eradicating sexual misconduct in surgery. The RCSEd aims to have a zero-tolerance approach to bullying, harassment and undermining and has produced a series of Professional Standards and an anti-bullying toolkit, e-module and other supporting materials. They also work with other healthcare partners on initiatives such as the Anti-Bullying Alliance, aimed at developing practical solutions to address bullying in the medical workplace.

In October 2024, the NHS introduced an anonymous reporting system for incidents of sexual abuse or misconduct, with a new framework outlining how those working within the health service should recognise, report and act on sexual misconduct in the workplace.

## Limitations

One possible limitation of this study was the 54.3 per cent response rate, and collecting more responses would enable a deeper understanding of the challenges faced by trainees – particularly for those reporting sexual assaults – and the associated impact on their mental health and wellbeing. One recommendation from the authors of this paper would be to run the survey again, publicising it more widely, to generate a higher response rate alongside a follow-up qualitative study to explore some of the issues in more detail. Replicating the study within other specialities, as trainees are a vulnerable group, is an option that should also be explored.

## Conclusions

Many respondents had experienced or witnessed a range of bullying, harassment and sexual harassment behaviours, yet very few (5 per cent or less) had reported them. Twenty-one per cent would not feel confident in reporting bullying/harassment or sexual harassment problems and 40 per cent do not feel safe raising concerns, mainly due to the fear of repercussions, being perceived as a difficult trainee/unable to cope, that no action would be/has been taken, that it is just what is expected as part of the job and doubts about confidentiality. Just 10 per cent said that the existing mechanisms for reporting problems are sufficient.

A number of initiatives have been introduced recently in the UK to address bullying and harassment, including a new duty for doctors to act, a zero-tolerance approach to sexual harassment and an anonymous reporting system for incidents of sexual misconduct. However, since existing reporting mechanisms are not felt to be sufficient by the respondents in this study, there is still potential for further development.
